# Endoscopic assessment of gastric emptying in older adults after preoperative administration of 5% glucose solution: a randomized controlled study

**DOI:** 10.1186/s12871-024-02847-5

**Published:** 2024-12-19

**Authors:** Yan Liu, Qian Yu, Run Wang, Linli Luo

**Affiliations:** 1 Department of Anesthesiology, Chengdu Jinniu District People’s Hospital (Sichuan Provincial People’s Hospital Jinniu Hospital), 389#, the Huazhaobi Zhongheng Street, Jinniu District, Chengdu, Sichuan 610041 China; 2 Department of Anesthesiology, Public Health Clinical Center of Chengdu, 18#, the Jingjusi Road, Jinjiang District, Chengdu, Sichuan 610041 China; 3 Department of Anesthesiology, West China Second University Hospital, Sichuan University, Chengdu, 610041 Sichuan P. R. China; 4 Key Laboratory of Birth Defects and Related Diseases of Women and Children (Sichuan University), Ministry of Education, Chengdu, 610041 P. R. China

**Keywords:** Carbohydrate, Elderly, Gastric emptying, Gastric volume, Endoscopy, Gastroscopy, Deep sedation, Preoperative fasting

## Abstract

**Background:**

Delayed gastric emptying of liquids may heighten the risk of aspiration reflux in elderly individuals. To investigate the gastric emptying of an oral supplement containing 5% dextrose solutions before sedation for gastroscopy.

**Patients and methods:**

A total of 100 elderly patients who were scheduled for elective gastroscopy were randomly assigned to two groups: the NPO(nil per os ) group and the dextrose solution ingestion group, which ingested a 5% dextrose solution (5 ml/kg) two hours before the procedure. The primary outcome measure was the gastric volume (GV) suctioned and measured during the gastroscopic examination. Secondary outcome measures included GV per weight (GV/kg), post-discharge blood glucose levels, patient discomfort assessed using the Visual Analog Scale (VAS), clarity of gastric mucosal visualization during gastroscopy, and the incidence of adverse events. Additionally, linear regression analysis was employed to identify factors influencing gastric volume.

**Results:**

There were no significant differences in gastric volume (GV) (*P*=0.258) and GV per weight (GV/W) (*P*=0.137) between the NPO group and the dextrose solution group. However,the NPO group had higher discomfort scores on the Visual Analog Scale compared to the dextrose solution group, with a statistically significant difference(*P*<0.001). The clarity of gastric mucosal visualization during gastroscopy was also significantly different between the two groups(*P*=0.038). Blood glucose levels and the incidence of adverse events showed no significant differences between the two groups.Multivariate linear regression analysis revealed that younger age and higher functional dyspepsia symptom diary (FDSD) scores were associated with larger gastric volume, with the regression equation being: GV = 79.922 - 1.186 $\times$ age + 0.556 $\times$ FDSD.

**Conclusion:**

In elderly patients, drinking 5 ml/kg of a 5 % glucose solution two hours prior to gastroscopy does not significantly increase gastric volume compared to midnight fasting.

**Trial registration:**

ChiCTR2100047031 (date of registration: 7 June 2021).

**Supplementary Information:**

The online version contains supplementary material available at 10.1186/s12871-024-02847-5.

## Introduction

The preoperative anaesthetic assessment is an essential part for anesthesia management.we do not simply focus on patients about to undergo surgical procedures in the operating theatre as there is an increasing number of procedures outside, such as endoscopy [1]. Preoperative evaluation of gastric emptying and gastric contents is crucial for assessing the risk of aspiration and planning for anesthesia management.Prolonged fasting before medical procedures can lead to metabolic disturbances induced by stress responses, such as hyperglycemia, insulin resistance, fat catabolism, and nitrogen loss, which are associated with adverse patient outcomes [2, 3]. The American Society for Gastrointestinal Endoscopy (ASGE) guidelines recommend the consumption of an appropriate amount of carbohydrates two hours before treatment for healthy patients with normal gastric emptying [4, 5]. However, in the elderly population, gastric electrical activity cell decline, rhythmic disturbances, and delayed gastric emptying have been observed [6–8]. Previous studies have shown that preoperative consumption of carbohydrate- containing clear fluids two hours before urological surgery does not increase the risk of aspiration [9–12]. Nevertheless, the evaluation of gastric emptying in that study relied on qualitative grading by gastric ultrasound, rendering the results unreliable [13, 14].

Gastroscopy is one of the main methods for early diagnosis, has been established as a secure and reliable method to assess the risk and severity of aspiration during induction of anesthesia [15]. Gastroscopy is a major methods for early diagnos, has been established as a secure and dependable method for evaluating fluid emptying, surpassing gastric ultrasound in this regard [13, 16, 17]. Precise observation and research on fluid gastric emptying in elderly patients undergoing gastroscopy can be achieved by direct aspiration and accurate fluid measurement under endoscopic visualization. However, limited clinical evidence exists regarding the potential risk of aspiration reflux associated with preoperative consumption of carbohydrate-containing clear fluids two hours before gastroscopy in the elderly population.The aim of this randomized controlled study is to evaluate the effects of preoperative consumption of 5% glucose solutions on gastric emptying, gastric fluid volume, and aspiration risk in older adults undergoing gastroscopy.We hypothesize that the preoperative intake of 5% glucose solutions up to two hours before gastroscopy does not significantly delay gastric emptying or increase gastric fluid volume and aspiration risk compared to traditional fasting, providing a safe and effective alternative for elderly patients.

## Methods

### Trial design and setting

This was a single center, randomized, blind controlled trial enrolled patients who underwent gas- troscopic examination from June 2021 to September 2022 and written informed consent was obtained from all subjects participating in the trial.This study was conducted at Jinniu District People’s Hospital. The study was approved by the Institutional Review Board of Ethics Committee of Jinniu District People’s Hospital (QYYKJ-2021-01). The trial was conducted in accordance with the Helsinki Declaration principles and registered with the Chinese Clinical Trial Registry (ChiCTR2100047031).The study was reported in line with the Consolidated Standards of Reporting Trials (CONSORT) guidelines.

### Patients’ assessment

A detailed assessment of the patients’ fasting and drinking history was conducted. Essential vital signs and fasting blood glucose levels were closely monitored, and patients’ discomfort levels concerning hunger, thirst, anxiety, and fatigue before the gastroscopic examination were evaluated using the Visual Analog Scale (VAS).Obtain each patients’ functional dyspepsia symptom diary score [18]. Organic gastric diseases was defined as oesophagitis, peptic ulcer disease, erosive gastritis, benign oesophageal stricture, Barrett oesophagus, or upper gastrointestinal malignancy,All other findings at endoscopy were classifed as functional gastric diseases [19]. The Sedative suitability of each patient was evaluated by a senior anesthetist using the ASA classification prior to gastroscopy.

### Selection criteria

The study included patients aged 60–80 years who were scheduled to undergo elective gastroscopy under general anesthesia and had an American Society of Anesthesiologists (ASA) physical status class of I or II. Exclusion criteria were: decline to participate, obesity(body mass index[BMI]>28kg/m2),diagnosed coexisting diseases that delay gastric emptying (e.g.,esophageal hiatal hernia,gastro-esophageal re- flux,ileus),diabetes,pregnancy, history of upper abdominal surgery including gastric surgery, psychiatric or mental disorders,use of any prokinetic drug up to 1 month previously.noncompliance or violation on the assigned protocol of preoperative fasting. All patients have signed written informed consent forms. Patients had the option to withdraw from the study upon request if they did not wish to continue.

### Random sequence generation, allocation, and blinding

A simple randomization method was employed to allocate patients into two groups.A random sequence was generated using computer-generated random numbers. Eligible patients were then randomly as- signed to two equal groups: Group N (nil per os group) consisted of patients who observed nil per os (NPO), and Group D(Dextrose solution group)comprised patients who consumed carbohydrate- containing clear fluids two hours before undergoing gastroscopy. The randomization process was con- ducted by a staff member with no involvement in patient care, follow-up, data collection analysis, or outcome assessment. To maintain blinding of patients’ group allocation, the randomly-generated numbers were concealed in sealed opaque envelopes. On the day before the gastroscopy, a research assistant sequentially opened the sealed envelopes to allocate participants to their respective groups. Throughout the study, the physicians performing gastroscopy and the researchers responsible for follow-up, data collection analysis, and outcome assessment remained unaware of the group assignments.

### Interventions

Both patient groups were allowed to consume solid and liquid food until midnight.Subsequently, the NPO group participants fasted from midnight until the gastroscopy.The dextrose solution group received instructions to consume a carbohydrate-containing beverage (dextrose solution; 5% carbo- hydrate, 5 kcal/100mL, 278 mOsm/kg) at a rate of 5ml/kg, two hours prior to the gastroscopic examination. Additionally, the dextrose solution group was restricted from consuming any fluids other than the designated carbohydrate beverage on the day of the gastroscopy.

### Anesthesia management

After admission to the examination room, standardized monitoring was conducted, including heart rate, blood pressure, and SpO$_{2}$. Subsequently, intravenous access was established, and the patient was positioned laterally for gastrointestinal endoscopy.Following adequate preoxygenation, anesthesia induction involved an intravenous injection of propofol (1-1.5mg/kg), sufentanil (0.025–0.05$\upmu$g/kg), and etomidate (0.05-0.1mg/kg). The gastroscopic examination commenced after the disappearance of the palpebral reflex. In cases where the heart rate remained >100 beats/min or increased by >20%, or if physical movement or coughing occurred, additional propofol (0.05-0.1mg/kg) was administered until smooth insertion of the gastroscope was achieved.Throughout the gastroscopy, propofol was administered as required to maintain a Ramsay score of 5 or 6, indicating a sluggish or no response to a glabellar tap or loud auditory stimulus. We recorded the changes in blood pressure and heart rate at four different time :basic vital signs (T$_{0}$), immediate endoscopic insertion (T$_{1}$), immediate endoscopic withdrawal (T$_{2}$), and patient leave off PACU (T$_{3}$).

### Gastric volume measurement

Upon entering the stomach, the endoscopic physician assesses the visual clarity of the gastric mucosa during gastroscopy. They then aspirate all the stomach contents and collect the aspirated liquid in a container for precise measurement by a data collector. Subsequently, the endoscopic physician conducts specialized examinations. Following the gastroscopy, the results of each patient’s upper gastrointestinal electronic endoscopy report will be documented. Based on the endoscopists’ diagnosis, the patients are categorized into functional gastric diseases and organic gastric diseases.

### Clarity of gastric mucosal visualization during gastroscopy

The endoscopic procedures were conducted by a single, experienced endoscopist who remained unaware of the patient’s group and premedication status. The endoscopies took place at a fixed period in Jinniu District People’s Hospital, using a video endoscope (EPK 1000 PENTAX, Japan). During the endoscopy, four distinct areas of the stomach, namely the antrum, the upper and lower parts of the greater curvature, and the gastric fundus, were individually evaluated for mucosal visibility. Each area was scored on a scale of 1 to 4 (in Supplementary Digital Content), known as the visibility score, based on a modified version of Kuo et al’s scoring system similar to the one used by Chang et al. [20]. The scoring criteria were as follows: (1) score 1, no adherent mucus on the gastric mucosa; (2) score 2, a small amount of mucus on the gastric mucosa without obstructing the view;(3) score 3, a large amount of mucus on the gastric mucosa, which could be cleared with less than 50 ml of water; and (4) score 4, a large amount of mucus on the gastric mucosa, requiring more than 50 ml of water to clear. The total visibility scores of all four areas were combined to obtain the Total Mucosal Visibility Score (TMVS) for each patient.

### Crisis event management

Gastroscopy, a commonly performed diagnostic procedure, can be associated with adverse events such as laryngospasm, regurgitation, aspiration, and circulation abnormalities. To ensure patient safety and prompt intervention, a comprehensive approach is adopted to manage these complications. Key strategies involve administering additional propofol and increasing oxygen flow to address laryngospasm, utilizing intravenous succinylcholine (50 mg) and conducting tracheal intubation for ventilation difficulties, implementing immediate oropharyngeal suctioning and Trendelenburg positioning in cases of regurgitation and aspiration. Circulation abnormalities are managed by administering atropine (0.3–0.5 mg) for low heart rate (<50 beats/min) and metaraminol (0.1–0.2 mg) for low blood pressure (<90/60 mmHg or a mean arterial pressure 20% lower than baseline). This comprehensive management approach is designed to mitigate risks and optimize patient outcomes during gastroscopy procedures.

### Study outcomes

Gastric volume (GV) is the primary outcome measure. Secondary outcome measures consist of gastric volume per body weight (GV/W), fasting blood glucose, blood glucose at 0.5 hours after ingestion of 5% dextrose solution and blood glucose at discharge from the post-anesthesia care unit. Additionally, patient discomfort is assessed using Visual Analog Scale (VAS) scores, and gastric mucosal visibility during gastroscopy is rated for clarity. The incidence of adverse events is also recorded.

### Sample size calculation

Based on the relevant literature and the preliminary results of our study (*N*=20), the mean gastric volume in the fasting group was found to be M=29.4 ml, while in the glucose solution group, it was M=16.9 ml. The standard deviations for gastric volume in the two groups were SD:17.3 and SD:11.4, respectively. Sample size calculation was performed using PASS 15.0 software, considering a significance level of $\upalpha$=0.05, a power of 1-$\upbeta$=0.8, potential data loss, and a maximum dropout rate of 20%. As a result, it was estimated that a total of *n*=100 patients would be included in both groups.

### Statistical analysis

Data analysis was conducted using IBM SPSS 25.0 statistical software. The normality of all quantitative data was assessed using the Shapiro-Wilk (S-W) test. Normally distributed data were presented as mean ± standard deviation (SD), and group differences were compared using the Student’s t-test. Non-normally distributed data were described as median (M$_{50}$) with interquartile range (P$_{25}$, P$_{75}$), and the Mann-Whitney U Wilcoxon test was employed for comparisons between groups. Qualitative data were expressed as frequencies and percentages [n (%)] and analyzed using appropriate statistical tests, including the chi-square test, continuity-corrected chi-square test, or Fisher’s exact test. During the gastroscopic examination, vital signs at predetermined time points were analyzed using repeated measures analysis of variance (ANOVA). The Bonferroni method, as implemented in SPSS, was applied to adjust for multiple within-group comparisons across time points and control the $\upalpha$ level. To identify factors influencing gastric volume, enter regression analysis was performed to establish a regression model. Statistical significance was set at *P*<0.05, and variables were included in the model regardless of their statistical significance.

## Results

**Fig. 1:**
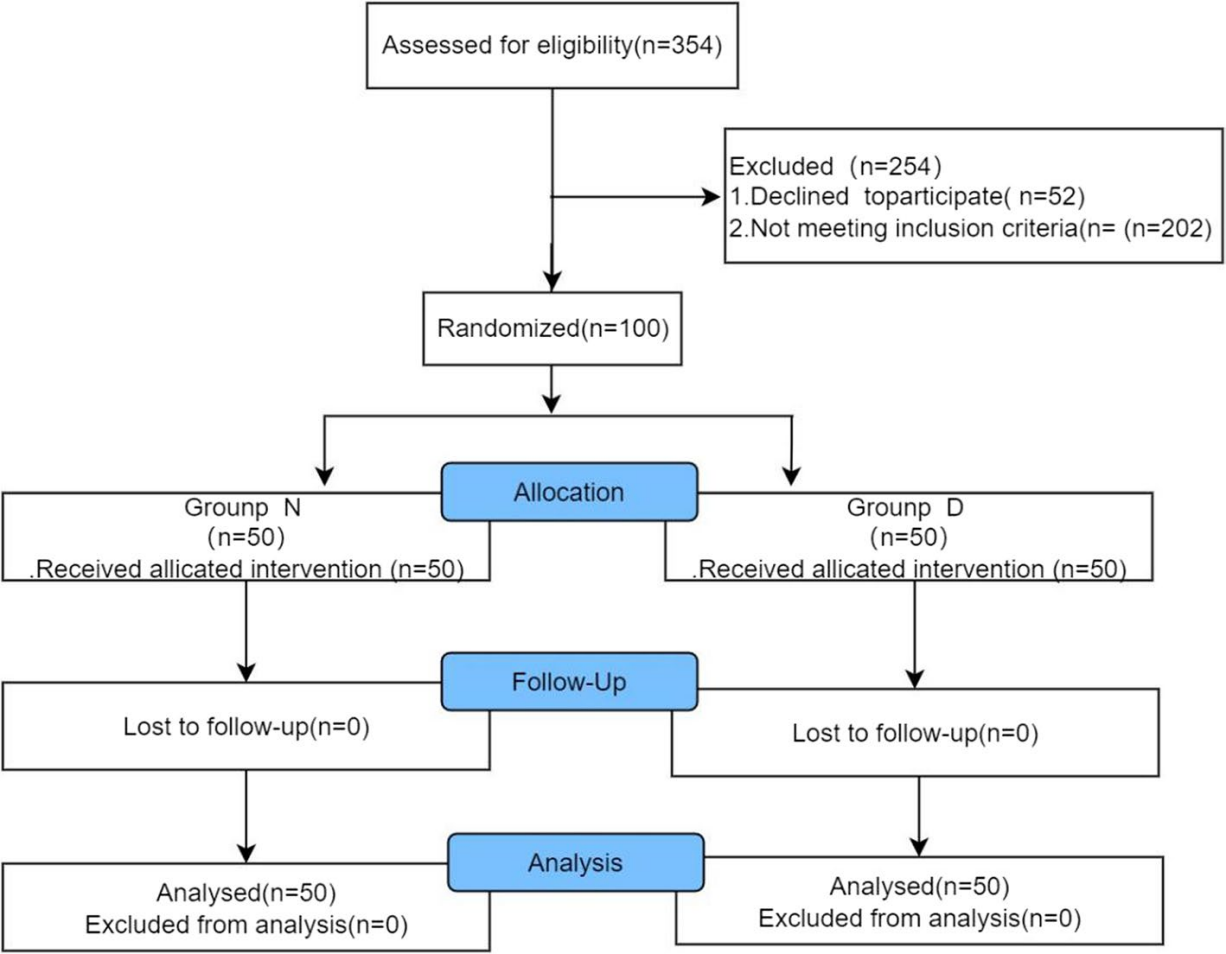
CONSORT flow chart illustrating patient selection and inclusion

This study commenced by screening a total of 354 patients. Among them, 254 individuals did not meet the inclusion criteria and were consequently excluded. Out of the 100 patients who participated in the trial, Ultimately, all participants adhered to the fasting instructions without experiencing any adverse side effects. Consequently, 100 individuals were eligible for inclusion in the statistical analysis (Fig.1). The baseline characteristics of the study cohort are presented in Table 1. No significant differences were observed between the NPO group and the dextrose solution group with respect to age, sex, height, weight, body mass index, ASA class, comorbidity, length of anesthesia, procedure, recovery time, or the presence of gastric diseases. The NPO group refrained from solid food intake for an average period of 13.6±2.6 hours, while the dextrose solution group observed a fasting period of 1.9±0.2 hours.

**Table 1:** Baseline characteristics of the patients

	Group N (n=50)	Group D (n=50)	*P-value*
Age (year)	66.0(64.0,71.25)	66.5(64.0,70.0)	0.855
Male( % )	17(34.0)	21(42.0)	0.41
ASA I/II	28/22	28/22	1.000
Mean height ( cm )	156 .9 ±6.9	158. 1 ±7.3	0.368
Weight (kg)	55.8±7.1	58.5±8.8	0.085
BMI ( kg/m2)	22.54±2.2	23.3±2.2	0.069
Scores of the FDSD gastic diseases	12.0(5.0,17.0)	13.0(7.0,20.0)	0.239
Organic gastric diseases ( % )	17(34.0)	14(28.0)	0.665
Functional gastric diseases ( % )	33(66.0)	36(72.0)	
Fasting time (hours)	13.6 ±2.6	1.9 ±0.2	< 0 .001
Length of anesthesia time (minutes)	7(5.75,8.25)	7(6,8)	0.955
Length of procedure (minutes)	5(4,7)	5(4,6)	0.402
Recovery time (minutes)	9( 5, 12.25)	9 (7, 12.50)	0.701
Sufentanil (ug)	2.0(2.0,2.63)	2.0(2.0,3.0 )	0. 369
Propofol dosage (mg)	75(70,85)	75(70,90)	0.703
Etomidate (mg)	4.0(4.0,5.0)	4.5(4.0,5.0)	0.121
Atropine (%)	1(2.0%)	1(2.0%)	1.00
Metaraminol (%)	7(14.0%)	3(6.0%)	0.20

### Primary outcome

**Fig. 2:**
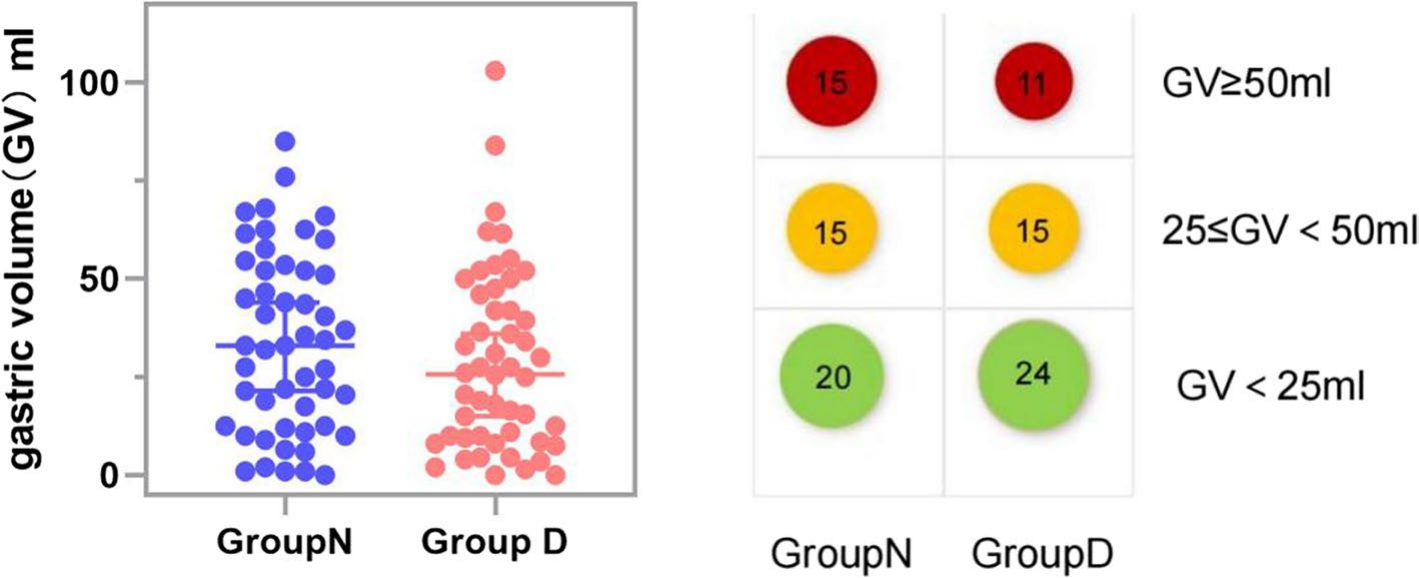
Boxplots of GV in group N (blue) and group D(red). The median values are presented as the thick black line in the middle of the box, The positions of the two ends correspond to the upper and lower quartiles of the data. The dots means each patient, GV= gastric volume, Group N= the nil peros group, Group D=dextrose solution group, Bubble Chart of gastric volume ,gastric volume categorized as >= 50ml, <50ml, <25ml, red, yellow, and green bubbles respectively to represent the number of people

The median gastric volume in the NPO group was 33.00 (12.38,52.38) ml, while it measured 25.75 (9.25,48.13) ml in the dextrose solution group, indicating a lack of statistical significance (*P*>0.05) (Table 2, Fig. 2). The median difference between these two groups amounted to 5.50 ml (*P*=0.258). Post hoc analysis demonstrated that the distribution of gastric content volume between two groups no statistical significance was observed (in Supplementary Digital Content).

**Table 2:** Comparison of gastric volume and gastric volume per body weight between two groups of patients

	Group *N* (*n*=50)	Group D (*n*=50)	*P-value*
Gastric Volume	33.00(12.38, 52.38)	25.75(9.25 , 48.13)	0.258
Gastric volume per body weight	0.58(0.22, 0.89)	0.43(0.18,0.76)	0.137

### Secondary outcome indicators

#### Gastric volume to body weight

**Fig. 3:**
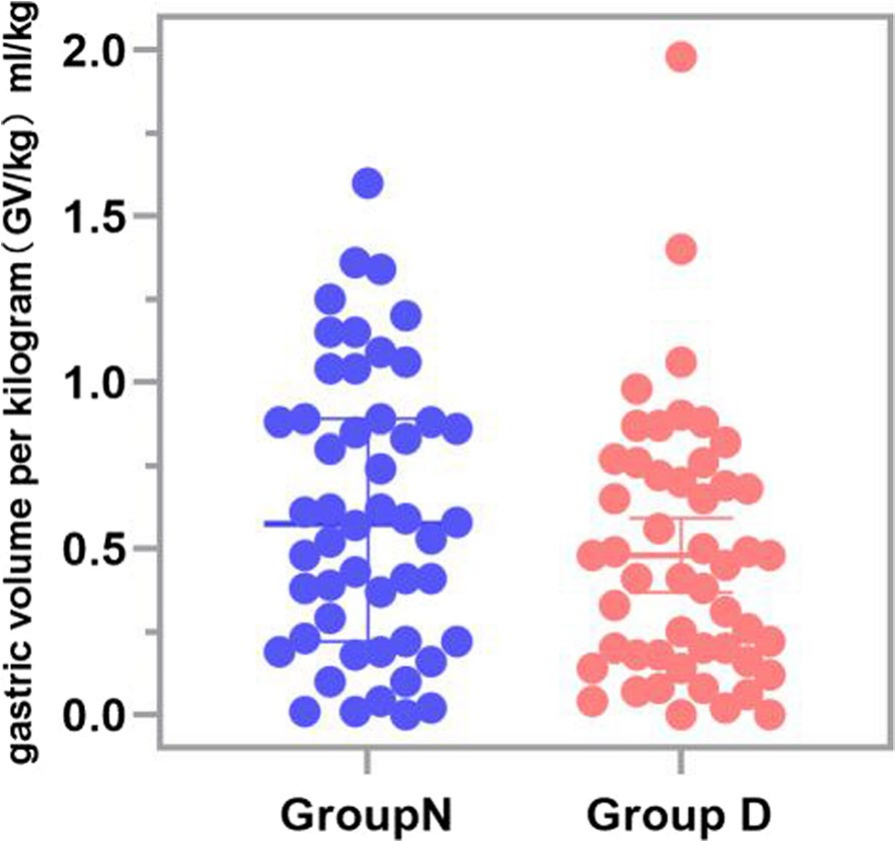
Boxplots of GV/kg in group N (blue) and group D(red). The median values are presented as the thick black line in the middle of the box, The positions of the two ends correspond to the upper and lower quartiles of the data, GV/W=gastric volume per weight

The median ratio of gastric content volume to body weight in the the NPO group stood at 0.58(0.22,0.89) ml/kg, compared to 0.43(0.18,0.76)ml/kg in the dextrose solution group(in Supplementary Digital Content), the median disparity between these groups was 0.13 ml/kg (*P*=0.137)and again, no statistical significance was observed (*P*>0.05) , Boxplots of gastric content volume to body weight in two groups are shown in Fig. 3.

#### Score for gastric mucosal visual field clarity

The median score for gastric mucosal visual field clarity during gastroscopy was 2 points in both the NPO group, as well as in the dextrose solution group(in Supplementary Digital Content).A statistically significant difference was observed between the two groups (*P*<0.05).

#### Discomfort rating

Detailed comparisons for hunger,thirst,and anxiety can be found in supplementary digital content.It’s worth noting that the hunger and thirst discomfort scores were higher in the NPO group and dextrose solution group compared to the dextrose solution group.

#### Post-discharge blood glucose leves

The median value of post-discharge blood glucose was 5.3(5.0,5.7)mmol/L in Group N,the median value of post-discharge blood glucose was 5.2(4.9,5.5)mmol/L in Group D, No statistically significant difference was observed between the two groups (*P*<0.05) (in Supplementary Digital Content).

#### Hemodynamic indicators

No statistically significant differences were observed in the changes in systolic blood pressure, diastolic blood pressure, mean arterial pressure, or heart rate between the two groups.Furthermore, during the gastroscopy examination, no adverse events, such as reflux and aspiration, were reported in either patient group (in Supplementary Digital Content).

#### Fitting a new model

Based on the data, several linear regression models were fitted to explore the relationship between suctioned gastric volume (GV) and potential predictors, including ASA grading, fasting for liquids, Scores of FDSD the gastic diseases, weight, age, and gender.The final model, selected as the best fit, included age and FDSD as significant predictors of GV. The regression equation is as follows:GV (mL) = 79.922 - 1.186 $\times$ Age (years) + 0.556 $\times$ FDSD.This model indicates that gastric volume decreases by approximately 1.186 mL for each additional year of age and increases by 0.556 mL for each additional Scores of FDSD the gastic diseases. The detailed model parameters and goodness-of-fit statistics are provided in Supplementary Digital Content.

## Discussion

This study utilized gastroscopy to quantify gastric fluid volume and found that administering a 5% dextrose solution at a rate of 5 ml/kg, given 2h ± 20minutes before gastroscopy in elderly patients, did not lead to a significant increase in gastric content volume. Additionally, subjective discomfort scores, including hunger and thirst, were notably lower, highlighting a potential improvement in patient comfort during the preoperative fasting period.

Another study [21] demonstrated that gastric contractions are nearly absent at blood glucose levels of 250 mg/dl, with significant reductions noted at acute blood glucose values of 140 mg/dl and 175 mg/dl. In our investigation, the group receiving the dextrose solution exhibited blood glucose fluctuations within the range of 8–10 mmol/L after consuming an average dose of 293 ml of a 5% dextrose solution over half an hour. While the 5% dextrose solution alleviated severe blood glucose fluctuations in elderly patients, transient inhibition of gastric emptying was observed in some individuals with higher blood sugar levels. However, no substantial difference in gastric volume was noted between the two groups, and the duration of gastric peristalsis inhibition during hyperglycemia was not precisely quantified. Importantly, a statistically significant difference was found in the variance between post-PACU blood glucose levels and fasting blood glucose levels in both groups. The dextrose solution group did not exhibit clinically significant reductions in blood glucose levels, and no patients experienced hypoglycemia. In contrast, the NPO group showed an upward trend in blood glucose levels post-gastroscopy, potentially due to extended fasting and the physiological stress response associated with gastroscopy. These findings suggest that 5% dextrose solutions can help maintain more stable blood glucose levels without increasing aspiration risk, providing a safe and beneficial alternative to prolonged fasting in elderly patients.

Our findings align with previous research, indicating that administering a 5 ml/kg glucose solution 2h±20minutes before endoscopic anesthesia and sedation does not significantly increase gastric content volume in elderly patients compared to fasting.Notably, 22% of patients in group D had a gastric volume greater than 50 ml, compared to 30% in group N, which was previously considered a threshold for reflux aspiration risk. In a study by José Eduardo de Aguilar Nascimento [22], ingesting 200 ml of a carbohydrate-rich nutrient formulation containing 67 g of carbohydrates and 8 g of whey protein 150 to 210 minutes before gastroscopy resulted in median gastric fluid volumes of 10 ml and 25 ml for the intervention group and control group, respectively. However, that study involved only 24 young patients with an average age of 35 years, limiting its applicability to elderly patients.

In assessing gastric emptying, various tools are available. Beyond the conventional gastric emptying scintigraphy [23], newer methods include gastric ultrasound, magnetic resonance imaging, intubation and aspiration via gastroscopy, and gastric bioelectrical impedance [24–26]. Among these, gastric ultrasound allows the observation of gastric emptying and motility but requires significant technical expertise due to challenges like gas interference. Magnetic resonance imaging and gastric bioelectrical impedance, while promising, are currently limited to research applications due to their high cost and operational complexity. Gastroscopy offers a practical alternative, allowing direct visualization and quantification of gastric contents. This makes it an excellent tool for verifying the safety of carbohydrate-containing drinks before general anesthesia for elective procedures, particularly in elderly patients.

The Functional Dyspepsia Symptom Diary (FDSD) is a novel measurement method focusing on patient-reported symptoms, including five main items (stomach burning, stomach pain, bloating, postprandial satiety, and early satiety) and three supplementary items (nausea, belching, and belching distress). FDSD scores have shown a positive correlation with functional dyspepsia-related scores [27]. Compared to other prediction models [14, 28, 29], this study’s regression analysis identified age and FDSD score as significant factors, influencing gastric content volume, offering potential clinical utility in predicting gastric emptying outcomes in elderly patients. We find that gastric volume decreases by approximately 1.186 mL for each additional year of age and increases by 0.556 mL for each additional Scores of FDSD the gastic diseases.These findings could guide preoperative preparation protocols, balancing safety and patient comfort in this vulnerable population.

## Limitations

This study has several limitations. Being a single-center trial, its findings may not be fully generalizable to other institutions with differing patient populations or clinical practices. The study included patients aged 60 to 80 years, with only 28.6% over 70 years. The exclusion of individuals over 80 years of age, due to potential challenges with cooperation, limits the applicability of the results to this subgroup, which may have distinct physiological characteristics and risk factors affecting gastric emptying and aspiration. Future studies should include this demographic to enhance generalizability.

The sample size was based on prior ultrasound studies and pilot experiments; however, discrepancies in pilot results led to an overestimation, reducing statistical efficiency. Additionally, gastric pH levels were not measured, despite prior evidence suggesting minimal impact from carbonated water consumption. The influence of anesthesia on gastric volume was also not evaluated, with the study assuming minimal short-term effects in line with institutional guidelines.

## Conclusion

Elderly individuals undergoing gastroscopy did not exhibit a notable rise in gastric volume subsequent to the oral consumption of 5 ml/kg of a 5% glucose solution within the 2-hour period prior to anesthesia induction. Furthermore, this intake did not heighten the susceptibility to reflux or aspiration, and it seemed to mitigate patient discomfort.

## Supplementary Information

Supplementary Material 1.

## Data Availability

No datasets were generated or analysed during the current study.
