# Supplementary figures and images for: Endoscopic assessment of gastric emptying in older adults after preoperative administration of 5% glucose solution: a randomized controlled study
Source: BMC Anesthesiol. 2024 Dec 19;24:458. doi: 10.1186/s12871-024-02847-5 (PMC11657266; doi:10.1186/s12871-024-02847-5)

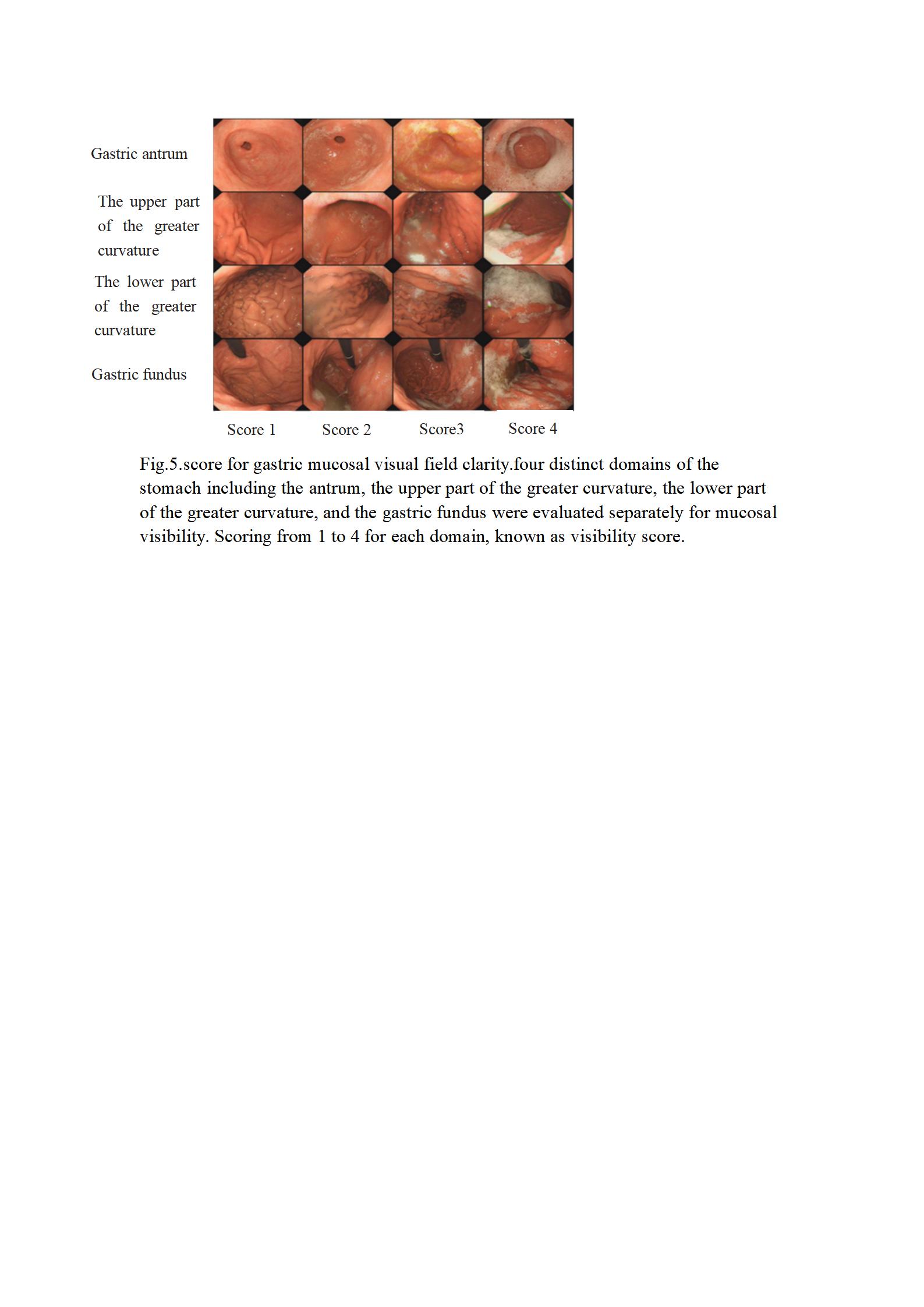

Supplement: Supplementary file 1 — Supplementary Material 1. [file 12871_2024_2847_MOESM1_ESM.zip › Fig.05.jpg]

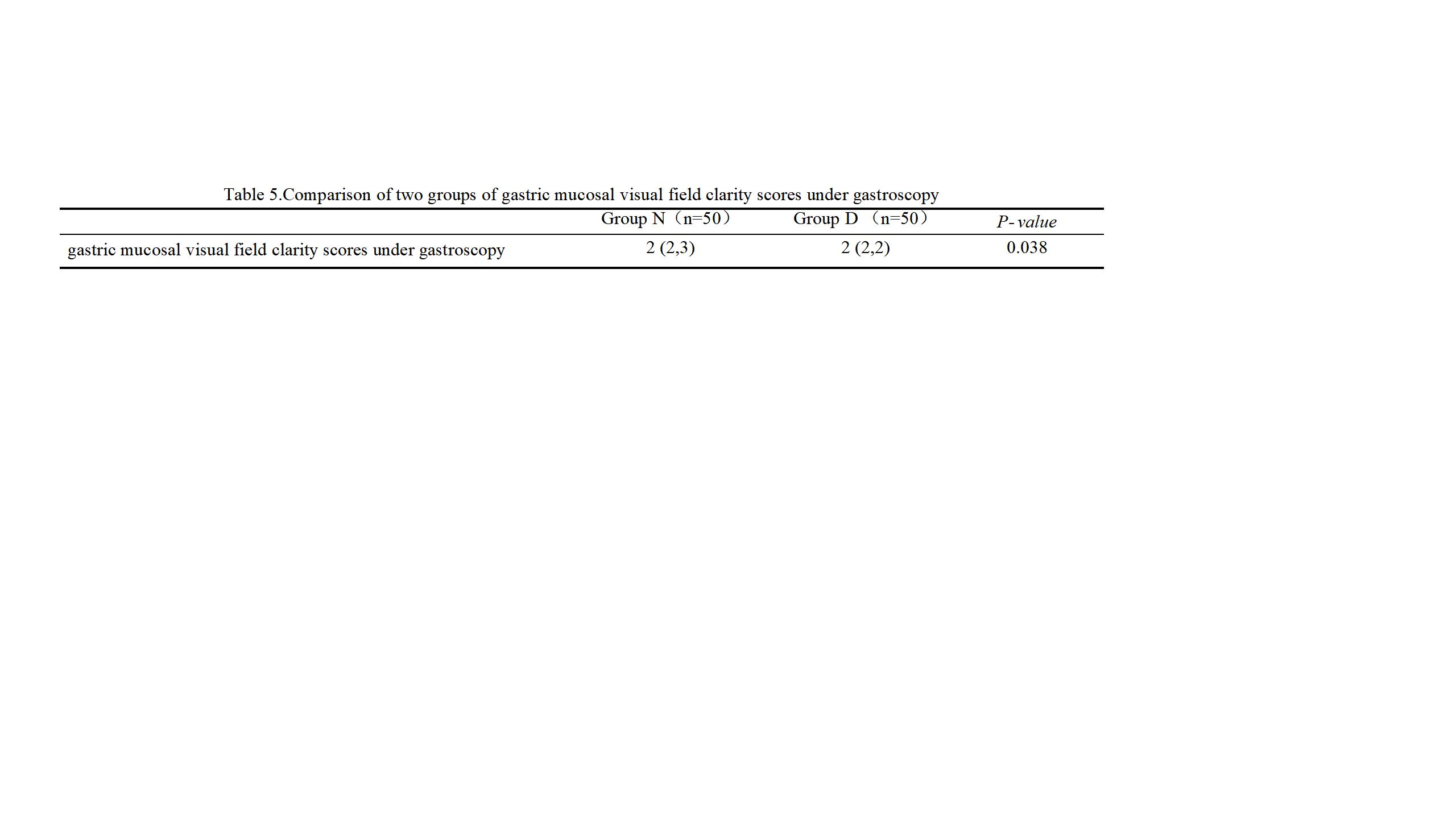

Supplement: Supplementary file 1 — Supplementary Material 1. [file 12871_2024_2847_MOESM1_ESM.zip › table_05.jpg]

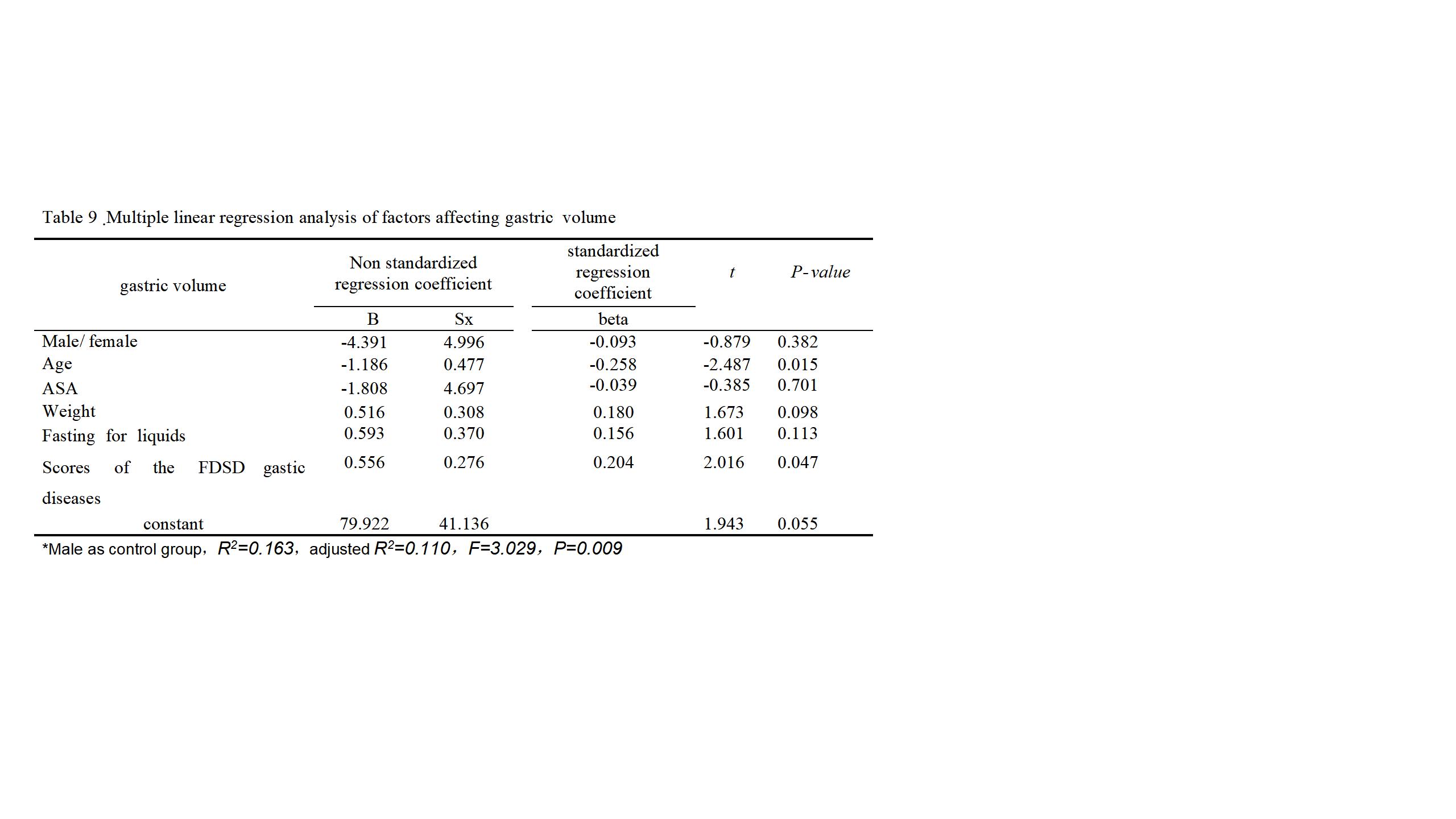

Supplement: Supplementary file 1 — Supplementary Material 1. [file 12871_2024_2847_MOESM1_ESM.zip › table_09.jpg]

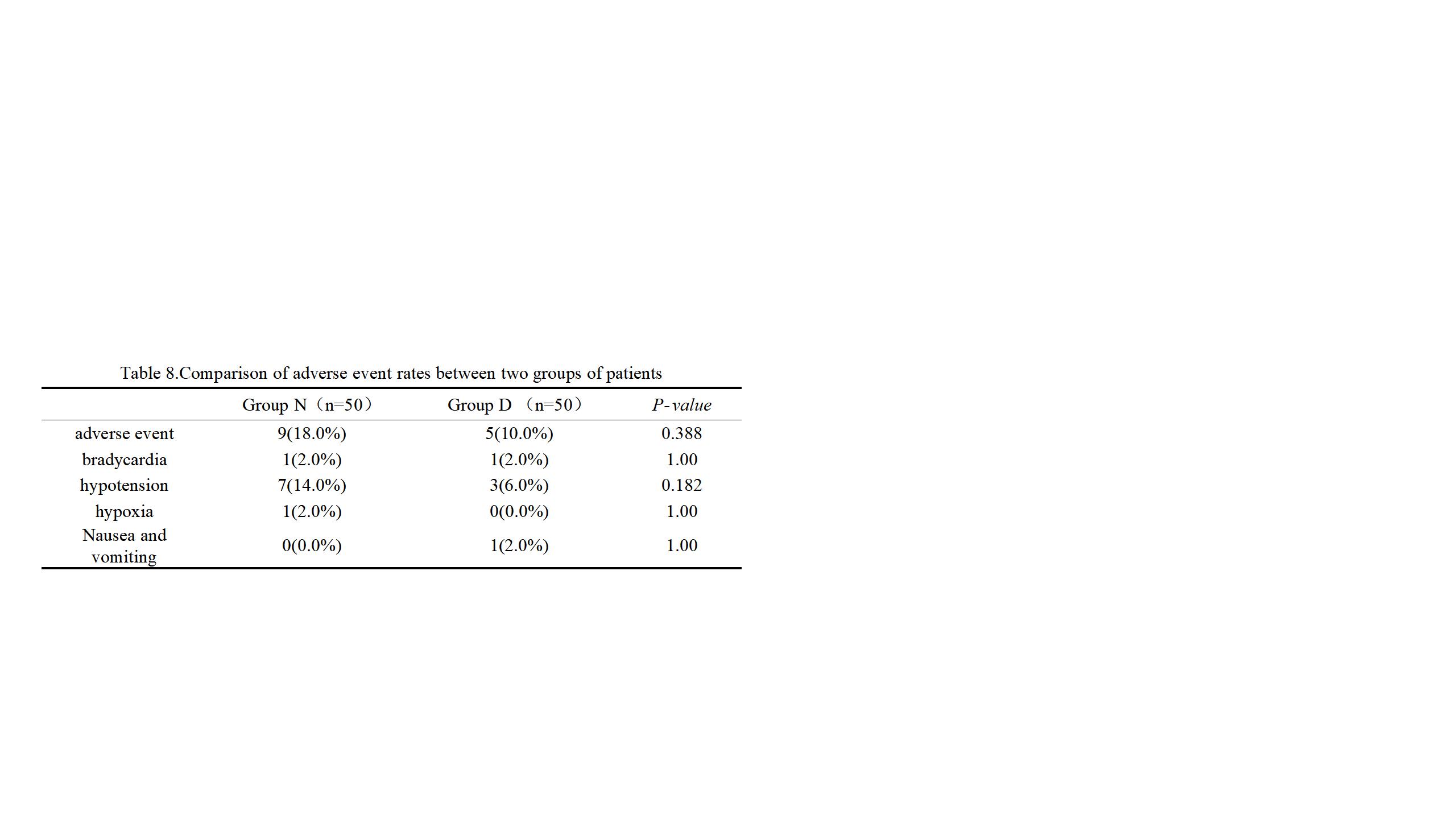

Supplement: Supplementary file 1 — Supplementary Material 1. [file 12871_2024_2847_MOESM1_ESM.zip › table_08.jpg]

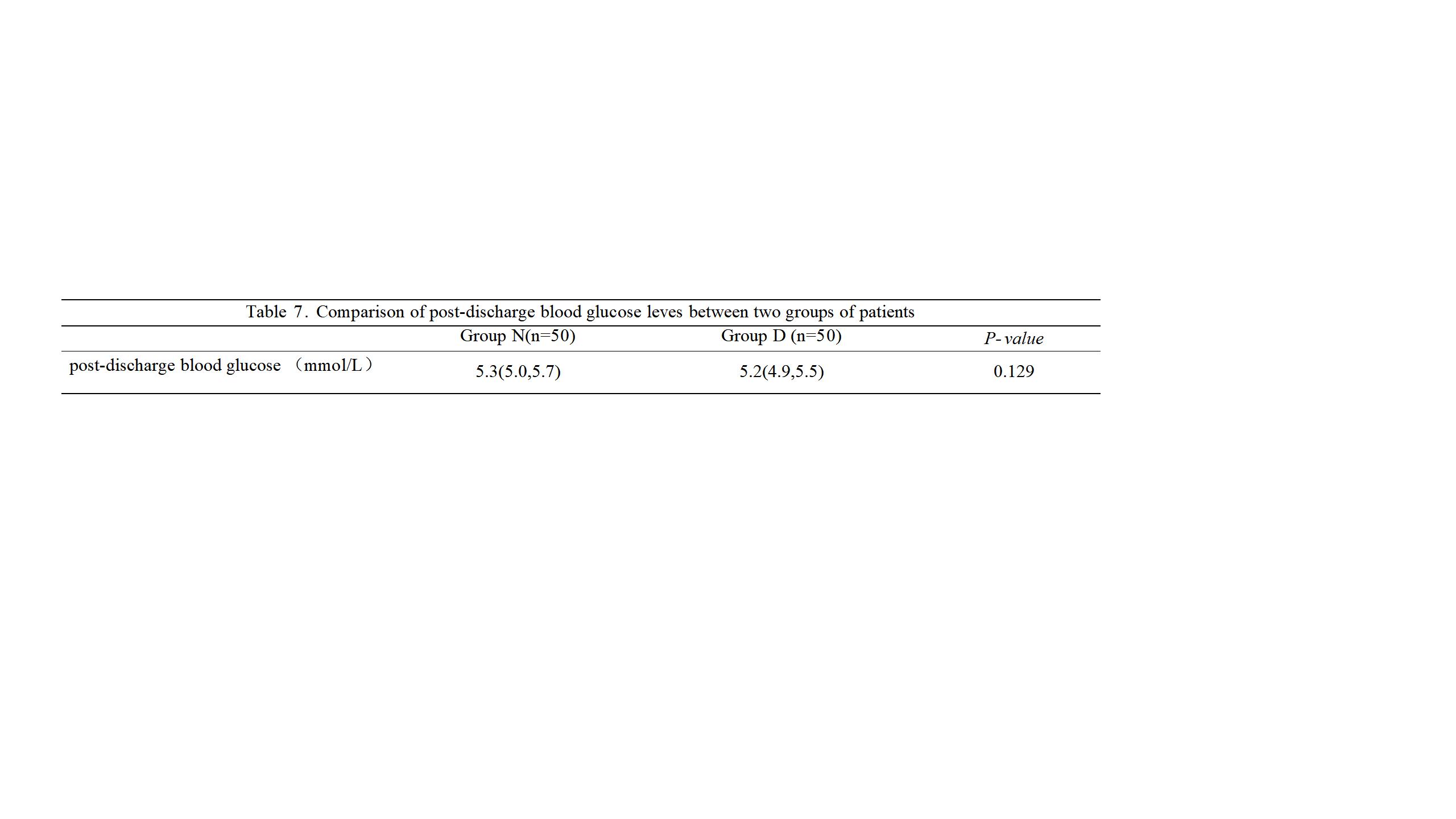

Supplement: Supplementary file 1 — Supplementary Material 1. [file 12871_2024_2847_MOESM1_ESM.zip › table_07.jpg]

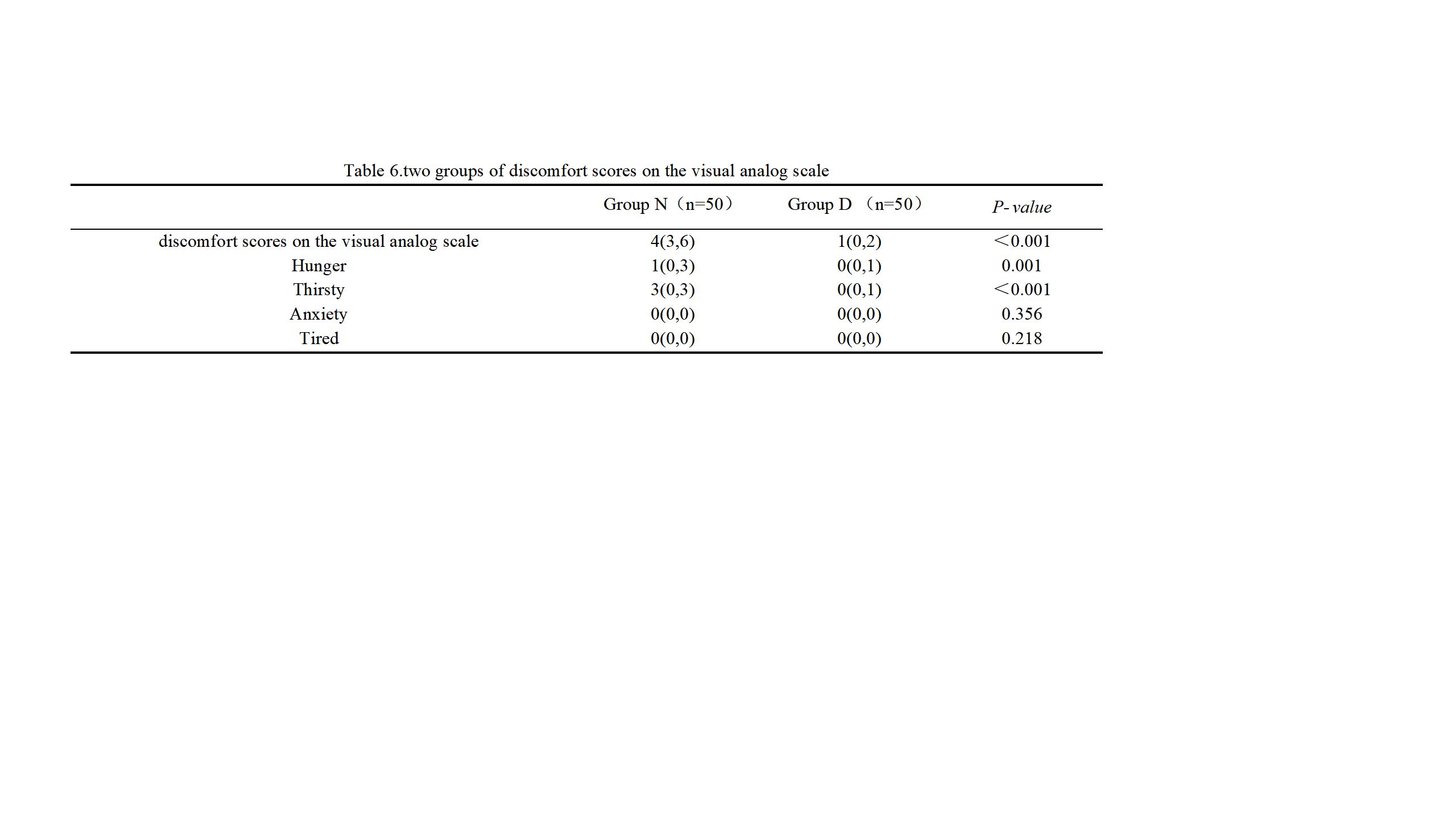

Supplement: Supplementary file 1 — Supplementary Material 1. [file 12871_2024_2847_MOESM1_ESM.zip › table_06.jpg]

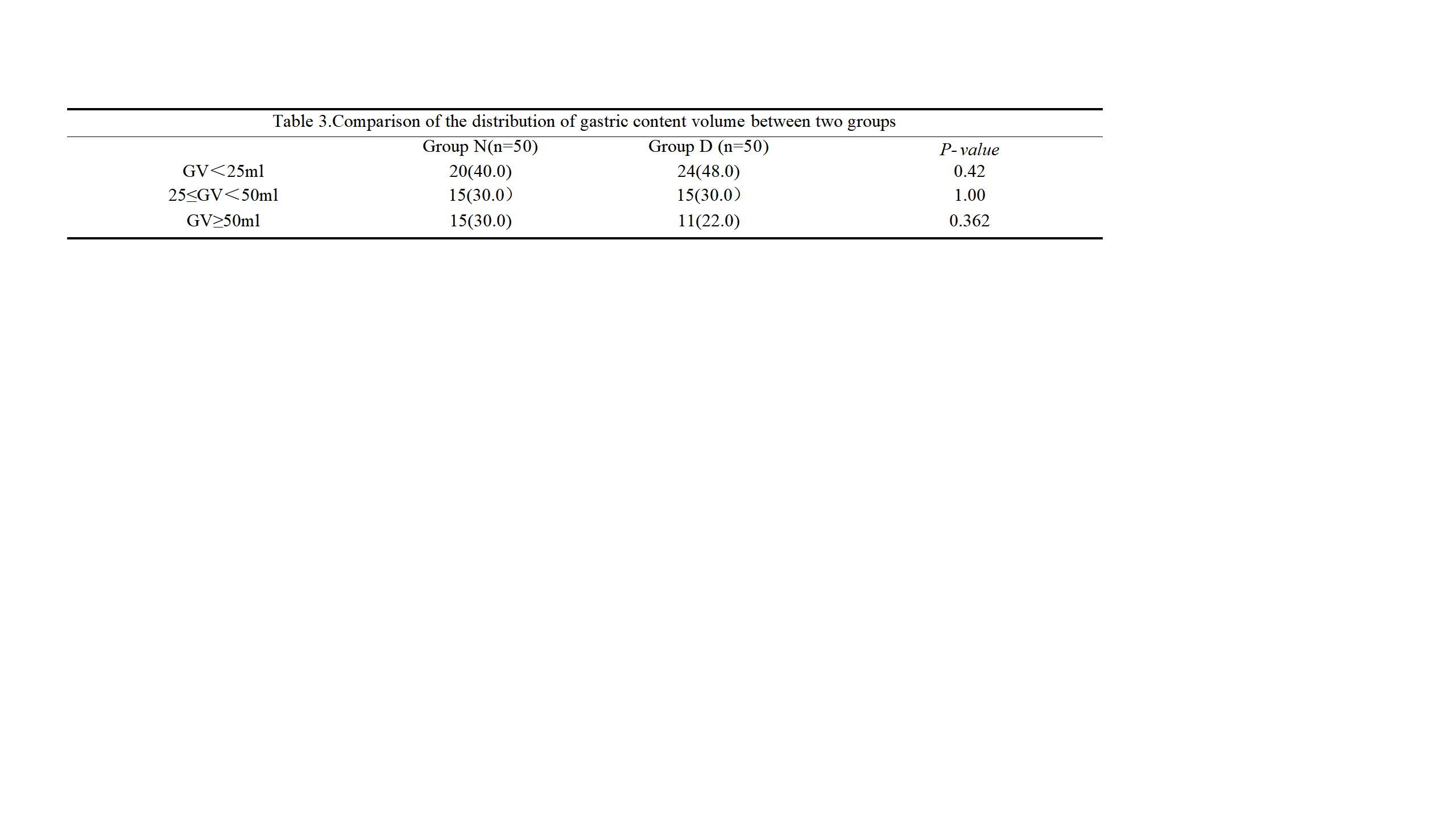

Supplement: Supplementary file 1 — Supplementary Material 1. [file 12871_2024_2847_MOESM1_ESM.zip › table_03.jpg]

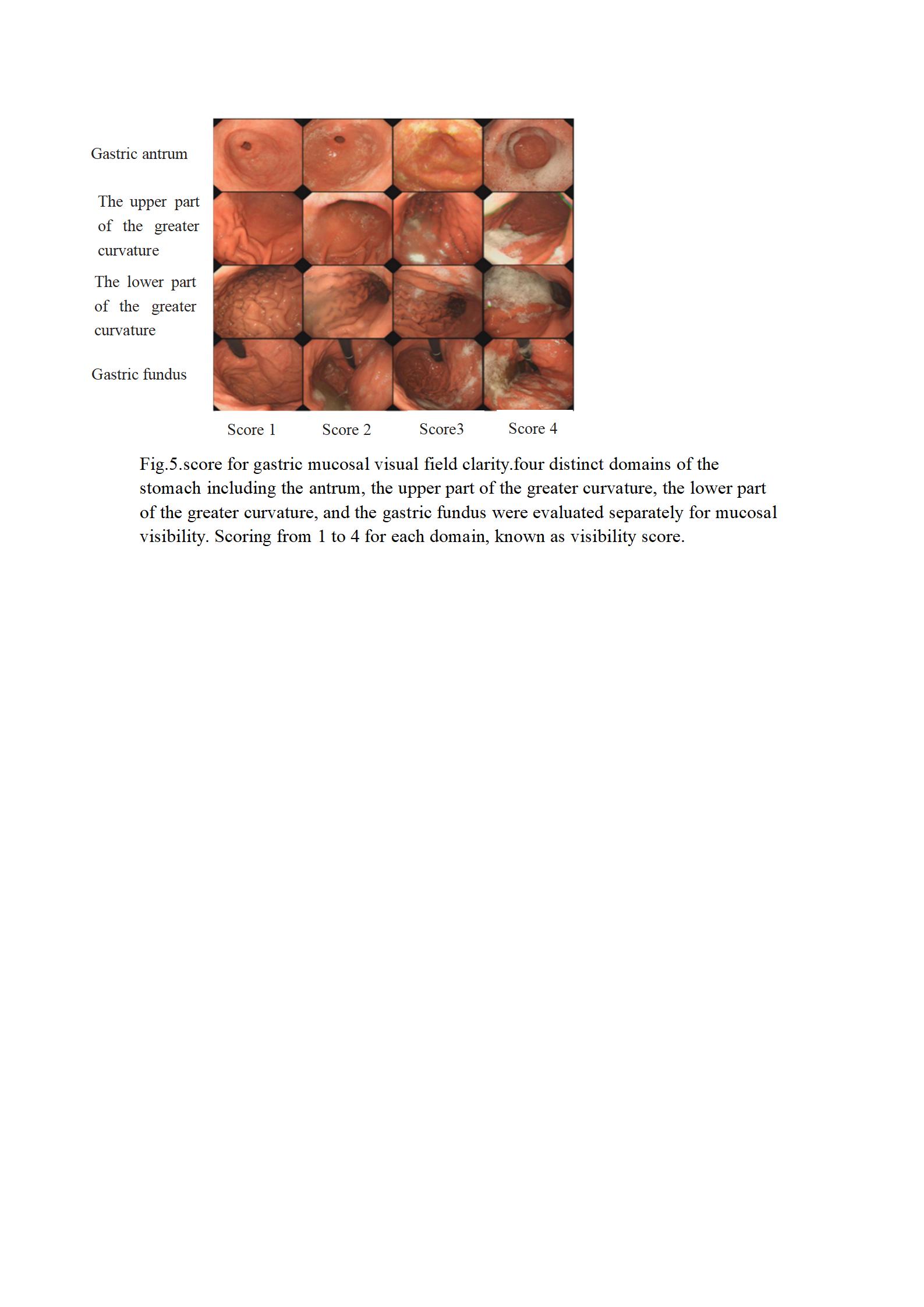

Supplement: Supplementary file 1 — Supplementary Material 1. [file 12871_2024_2847_MOESM1_ESM.zip › Fig.6.jpg]
